# Prevalence and factors associated with immunization coverage among children under five years in Mohamed Mooge health center, Hargeisa, Somaliland: a cross-sectional study

**DOI:** 10.1186/s12887-023-04371-w

**Published:** 2023-10-30

**Authors:** Mohamed Mussa Abdilahi, Ahmed Ismail Mohamed, Kiruja M. Jonah, Abdisamad Shukri Ismail

**Affiliations:** College of Medicine and Health Science, University of Hargeisa, Hargeisa, Somaliland

**Keywords:** Vaccine Preventable Diseases, Immunization coverage, Under five children, Somaliland

## Abstract

**Background:**

Routine immunization contributes greatly to reduction in mortality from vaccine preventable diseases among children. The Somaliland Demographic and Health survey, 2020 revealed that only 13.7% of children in Marodijeh (Hargeisa) region had received all recommended vaccines, which is far below the World Health Organization (WHO) target of 80%. We therefore, assessed factors associated with immunization coverage among children under five years at Mohamed Mooge Health Center in Hargeisa, Somaliland.

**Methods:**

Institutional based cross-sectional study was conducted on 174 systematically sampled, consented mothers that visited Mohamed Mooge Health Center for antenatal care during December 2022 to May 2023. Data was collected using a structured questionnaire. Data was analysed using SPSS and the relationship between dependent and independent variables was checked chi-square test at p ≤ 0.05. Finally, candidate variables were tested by using multivariate logistic regression in order to control potential confounders and the result was presented using AOR and 95% confidence interval. Model fitness was checked using Hosmer-Lemeshaw goodness of fit test with P > 0.05 for fitness. Multicollinearity between variables was checked using correlation coefficients at 0.80 or higher.

**Result:**

Among 174 study participants, the prevalence of overall vaccine completion in this study was 55.3%. Women who had being aware about childhood immunization on BCG vaccination (AOR = 3.887; 95% CI: 1.275, 6.844), pentavalent (AOR = 11.385; 95% CI: 5.424–14.464), and measles (AOR = 3.074; 95% CI: 1.822–6.130) had higher odds of having immunized their children. Mothers who had employment had higher odds of having their children immunized against measles (AOR = 4.069; 95% CI: 1.822–6.130) compared to those who had not.

**Conclusions:**

Full immunization coverage was lower than the target set by the World Health Organization in this study area. The current study revealed that, the mother’s awareness of childhood vaccinations on BCG, pentavalent, measles and employment status of mothers were positively associated with immunization coverage. To promote vaccination coverage, the government should implement a national awareness campaign on childhood immunization and increase the number of outreach services.

## Background of the study

The World Health Organization (WHO) defines immunization as the process whereby a person is made immune to an infectious disease. Immunization is one of the most cost-effective measures in public health to protect children from serious diseases and also prevent the spread of those diseases to others [1]. An expanded program on immunization (EPI) was introduced by the World Health Organization (WHO) in 1974 to develop and expand immunization programs worldwide to reduce child morbidity and mortality [2, 3].

Globally, the level of morbidity and mortality from vaccine preventable diseases have decreased in recent years due to improvement of childhood vaccinations. Vaccine preventable diseases are still responsible for 1.5 million deaths each year among children who are under-five years old. Immunization was reported to have saved around two to three million lives of children at the end of the year of 2011 [4, 5]. It is also reported that around 27 million children who are less than one year old were reported not vaccinated, especially measles in 2007. It also estimated that globally, around 5.2 million children aged one to 59 months die, of which 29% are deaths due to vaccine preventable diseases [6].

Deaths related to complications of measles were reduced by 74% worldwide and by 89% in sub-Saharan Africa (SSA) for seven years from the year 2000 to 2007 [5]. Around 19.9 million of children who are one year of age did not get routine immunization services such as; pentavalent, measles and polio; about 60% of these children from the above figure have been living in the following 10 countries; India, South Africa, Ethiopia, Democratic Republic of Congo, Angola, Nigeria, Indonesia, Iraq, Afghanistan, and Pakistan [7]. Based on the huge number of published literatures around the factors associated with immunization coverage, age of mother, child’s sex, maternal educational status, possession of immunization card, accessibility related issues, concern about availability of vaccines and many other predictors may contribute towards the likelihood of fully immunizing the children [8–11].

The Somaliland demographic health survey 2020 (SHDS-2020) showed about 13% of children aged between 12 and 23 months were fully vaccinated, while 68% of children had not received any vaccination. With regard to specific vaccines, 31% of children under-five had received BCG vaccine, 32% had pentavalent 1, 15% pentavalent  2, and 13% of children had received Pentavalent3. On the other hand, 32% of children had received polio 0 vaccines, 32% had received polio 1 vaccines, 16% had received the polio 2 vaccine and 15% of children had received polio 3 vaccines. On the other hand, around 15% of children had received measles vaccination at any time before the survey [12].

The survey also showed the region that has received the highest percentage of all basic vaccines was Sahil region and was 22.9%, Togdheer 14.7%, Marodijeh 13.7%, Sanaag 10.4%, Awdal 8.9% and Sool region was 8.4% [12].

In order to increase child immunization coverage, the underlying causes and parent’s reasons of not immunizing their children should be known. In the study area, so far there is a paucity of evidence on immunization coverage among under five years children. Therefore, this study aimed to address the prevalence and associated factors with immunization coverage among under five children at Mohamed Mooge Health Centre in Hargeisa, Somaliland. It will help policy makers, program implementers and service providers to eliminate the obstacles and improve child immunization coverage in order to attain the intended prevention and control of vaccine preventable diseases. It will also serve as a baseline study for future studies.

## Materials and methods

### Study design, study setting and study period

An institutional-based cross-sectional study was conducted from December 2022 to May 2023 among mothers whose children are under five years of age. The study was conducted at Mohamed Mooge Health Center in Mohamed Mooge district that is located south-eastern part of the Hargeisa which is the capital city of Somaliland. Mohamed Mooge Health Center (MMHC) is the largest health facility among four health centers in the district and provides wide range of health services including ANC, PNC, delivery, extended program of immunization (EPI) and nutritional programmes. Approximately 500 of under-five children attend for immunization per month.

### Population of the study

The source of population was all mothers with under five year children who were attending Mohamed Mooge Health Center.

### Inclusion and exclusion criteria

All mothers whose children were equal or less than 5 years and were in the Health Center during data collection process were included in this study. Those participants who did not give consent or not willing to participate in this study were excluded from the study.

### Sample size determination

The sample size required was determined based on single proportion population formula with the assumption of 5% margin of error, 95% confidence level, and 13% of vaccinated children in Somaliland [12] which is taken from Somaliland Health and Demographic Survey report 2022 (SLHDS) to get the possible sample size of this study. The sample size was calculated as follows:

$$\begin{array}{l}{\bf{Sample}}{\rm{ }}{\bf{size}}{\rm{ }}\left( {\bf{n}} \right) = \frac{{(Z\alpha /2) \times p(1 - P)}}{{{{(d)}^2}}},\\(n) = \frac{{{\rm{(1}}{\rm{.96)}} \times {\rm{0}}{\rm{.13(1 - 0}}{\rm{.13)}}}}{{{{(0.05)}^2}}} = 174\\\end{array}$$

**Where**.

i. n is the calculated sample size.
ii. P prevalence of previous studies (13%).
iii. Z a/_2_ is the value of standard normal distribution (Z- statistic) at the 95% confidence level ((α 0.05) which is 1.96.
iv. d is te marin of error 5% (0.05).

### Sampling techniques

The total number of under-five children who attend EPI service for this study was assumed to be similar to the total number of EPI attendants of the month prior to this study period and it was 522 attendants. So, we divided 174 (sample) by 522 (sampling frame) so that a sampling interval (k) was 3.

To determine the first respondent, simple random sampling technique among the first three participants was used. Therefore, starting from the first respondent, every 3rd participant was then included until we got the required sample size.

### Study variables

In general, the dependent variable (outcome) of this study was, whether the child is fully vaccinated or not, however we have examined main vaccines separately as each has its own factors that may contribute to the child not getting fully immunized. Socio-demographic profile of women *(Age, educational level and occupational status);* vaccine availability factors *(waiting time, motivation to come back and ever miss vaccine)*; and accessibility related factors (mother’s awareness and distance to the health facility) was independent variables of the study.

### Operational definitions

- **Complete vaccination**: a child who received nine basic vaccines is considered as complete vaccination.
- **Incomplete vaccination**: a child who received some of the vaccines and/or not full dose of nine basic vaccines is considered as incomplete vaccination.
- **BCG**: A vaccine containing bacillus Calmette-Guerin (BCG), an attenuated strain of Mycobacterium bovis, with non-specific immunoadjuvant and immunotherapeutic activities.
- **OPV**: Oral taking attenuated vaccine that used to prevent against polio infection.
- **Pentavalent**: Is comprehensive vaccine used to protects against five diseases (diphtheria, tetanus, pertussis, hepatitis B and Haemophilus influenzae type B).

### Data collection and analysis

Data were collected using a structured questionnaire. Data collection was facilitated by three qualified nurses. Data collectors was trained for one day on collecting accurate and reliable data by the principle investigator. The questionnaire was adapted from different relevant published literature and then contextualized to the local situation [13]. The questionnaire was pretested on 5% of the sample that haven’t be included in the main study. Data collected include socio-demographic factors, availability, and accessibility. Data collected each day were checked visually for completeness by the supervisors and the principal investigator. Then, the investigators entered the data into SPSS version 20 software for cleaning and analysis, missing data was handled by using imputation method in the software. Descriptive statistics were used to describe data. Bivariate analysis was employed to identify factors associated with immunization status among children under five. During bivariate analysis, association between categorical variables were assessed using the Chi square test at 95% Confidence Interval (CI). A Multivariable logistic regression model with full immunization status as dependent variable was built to rule out possible confounders. All analyses were done at 95% CI and 0.05 of significance.

## Results

### Prevalence of immunization coverage

**Fig. 1:**
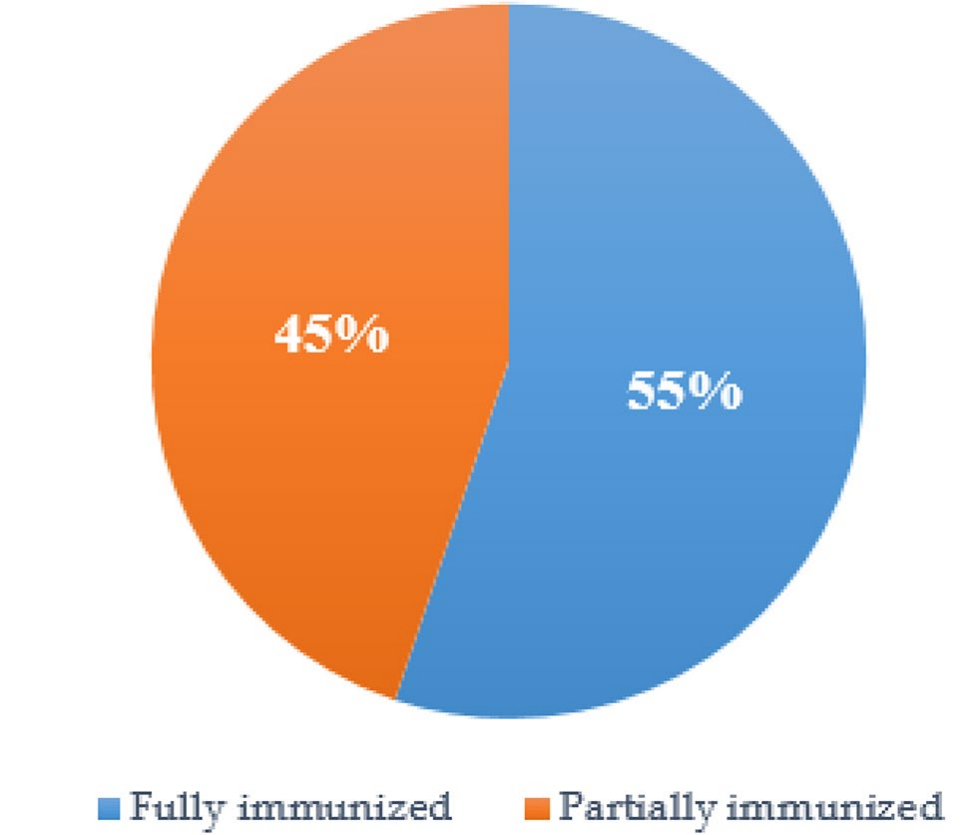
The prevalence of immunization coverage among children under five years in Mohamed Mooge Health Center, Hargeisa, Somaliland, 2023 (n = 174)

In this study, the prevalence of immunization coverage was assessed by asking several questions about the doses, schedule and types of main vaccines. Then, combination and recording these questions was used to determine whether the child is fully immunized or nor. The result showed that 55% of children were fully immunized. (Fig. 1).

### Factors associated with immunization coverage

To determine possible predictor variables associated with immunization coverage, chi-square test was done to find out candidate variables for multivariate regression and result was presented using p value and adjusted odds ratio (AOR).

#### Sociodemographic characteristics of study participants

The result revealed; educational level of mothers shows significant association with BCG (p < 0.0001) and OPV *(p* < 0.0001). The study also shows that; the occupational status of mothers was significantly associated with receiving BCG (*p* = 0.024), and measles (*p* = 0.002) vaccines. Furthermore, place of delivery was related with taking BCG (*p =* 0.045) and OPV (*p =* 0.037) vaccines. (Table 1).

**Table 1:** Sociodemographic characteristics of child mothers/caretakers on immunization coverage among children under five years in Mohamed Mooge Health Center, Hargeisa, Somaliland, 2023 (n = 174)

Variables	BCG		P value	OPV		P value	Pentavalent		P value	Measles		P value
	Yes n= 171%	No n= 3%		Yes n= 83%	No n= 91%		Yes n= 137%	No n= 37%		Yes n= 67%	No n= 107%	
**Age of the Mother’**			0.269			0.163			0.054			0.606
<20 20-29 >30	23(13.5%) 70(40.9%) 78(45.6%)	1(33.3%) 0(0.0%) 2(66.7%)		14(16.9%) 38(45.8%) 31(37.3%)	13(13.9%) 32(34%) 49(52.1%)		16(11.7%) 61(44.5%) 60(43.8%)	8(21.6%) 9(24.3%) 20(54.1%)		8(11.9%) 30(44.8%) 29(43.3%)	16(15%) 40(37.3%) 51(47.7%)	
**Educational**												0.522
Formal education None formal education No education	86(50.3%) 42(24.6%) 43(25.1%)	1(33.3%) 1(33.3%) 1(33.3%)	1.000	26(31.3%) 38(45.7%) 16(19.20%)	61(64.9%) 26(27.7%) 7(7.4%)	0.000	72(52.5%) 33(24.1%) 32(23.4%)	15(40.5%) 10(27.1%) 12(32.4%)	0.401	33(49.3%) 23(34.3%) 11(10.4%)	54(50.5%) 41(38.3%) 12(11.2%)	
**Occupation**			0.024			0.718			0.082			0.002
Employed Unemployed	110(64.3%) 61(35.6%)	1(333%) 2 (66.6%)		9(11.2%) 71(88.8%)	9(9.6%) 85(90.4%)		17(12.4%) 120(87.6%)	1(2.7%) 36(97.3%)		13(19.4%) 54(80.6%)	5(4.7%) 102(85.3%)	
Delivery place			0.029			0.037			0.004			0.665
Home Health facility	16(9.4%) 155(90.6%)	2(66.7%) 1(33.3%)		9(11.2%) 71(88.8%)	22(23.4%) 72(76.6%)		9(6.6%) 128(93.4%)	9(24.3%) 28(75.7%)		13 (19.4%) 54(80.6%)	18(16.8%) 89(83.2%)	

#### Availability related factors of study participants

The current study found that, the waiting time was significantly related with receiving BCG (*p =* 0.014), OPV (*p =* 0.045), Pentavalent (*p =* 0.038), and Measles (*p =* 0.003). Motivation to come back to the health care facility and BCG (*p =* 0.059), OPV (*p =* 0.069), pentavalent (*p =* 0.098) and measles (*p =* 0.029) were statistically associated (Table 2).

**Table 2:** Availability related factors of child mothers/caretakers on immunization coverage among children under five years in Mohamed Mooge Health Center, Hargeisa, Somaliland,2023 (n = 174)

Variables	BCG		P value	OPV		P value	Pentavalent		P value	Measles		P value
	Immunized n= 171	Not immunized n= 3		Immunized n= 80%	Not immunized n= 94%		Immunized n= 137%	Not immunized n= 37%		Immunized n= 67%	Not immunized n= 107%	
Ever miss a vaccine			0.297			0.348			0.922			0.683
Yes No	61(35.7%) 110(64.3%)	2(66.7%) 1(33.3%)		26(32.5%) 54(67.5%)	37(39.4%) 57(60.6%)		33(24.1%) 104(75.9%)	7(18.9%) 30 (81%)		23(34.3%) 44(65.7%)	40(37.4%) 67(62.6%	
Waiting Time			0.022			0.045			0.038			0.003
Less time Prolonged time	143(83.6%) 28(16.4%)	1(33.3%) 2(66.7%)		45(56.2%) 35(43.8%)	57(60.6%) 37(39.4%)		116(84.7%) 21(15.3%)	28(75.7%) 9(24.3%)		36(53.7%) 31(46.3	66(61.7%) 41(38.3%)	
Motivation to come back			0.206			0.049			0.072			0.029
Yes No	122(71.3%) 49(28.7%)	1(33.3%) 2 (66.7%)		62(77.5%) 18(22.5%)	61(64.9%) 33(35.1%)		101(73.7%) 36(26.3%)	22(59.5%) 15(40.5%)		52(77.6% 15(22.4%)	71(66.4%) 36(33.6%)	

#### Accessibility related factors of study participants

Regarding the accessibility related factors, the study finds that, the women’s level of awareness is significantly associated with receiving of BCG (*p* = ≤ 0.001), OPV (*p* = 0.019), Pentavalent (p = ≤ 0.001) and Measles (*p* = 0.010). No statistical association observed between distance from health care facilities and BCG, OPV, pentavalent and measles (Table 3).

**Table 3:** Accessibility related factors of child mothers/caretakers on immunization coverage among children under five years in Mohamed Mooge Health Center, Hargeisa, Somaliland,2023 (n = 174)

Variables	BCG		P value	OPV		P value	Pentavalent		P value	Measles		P value
	Yes n= 101%	No n= 73%		Yes n=80%	No n= 94%		Yes n=137%	No n= 37%		Yes n= 67%	No n= 107%	
Distance			0.080									0.347
≤5 km >5 km	52 (51.5%) 49(48.5%)	45 (61.6%) 28(38.4%)		10(12.5%) 70(87.5%)	18(19.1%) 76(80.9%)	0.234	117(85.5%) 20(14.5%)	27(73%) 10(27%)	0.364	13(19.4%) 54(80.6%	15(14%) 92(86%)	
Awareness			0.042			0.019			0.000			0.010
Yes No	151(88.3%) 20(11.7%)	1(33. 3%) 2(66.7%)		75(93.8%) 5(6.2%)	77(81.9%) 17(18.1%)		129(94.2%) 8(5.8%)	23(62.2%) 14(37.8%)		47(70.1%) 20(29.9%)	88(82.2%) 19(17.8%	

### Multivariate regression analysis of factors associated with immunization coverage among children under five years in Hargeisa, Somaliland, 2022 (n = 174)

In the multivariate analysis, being aware about childhood immunization were higher odds of BCG vaccination (AOR = 3.887; 95% CI: 1.275, 6.844), pentavalent (AOR = 11.385; 95% CI: 5.424, 14.464), and measles (AOR = 3.074; 95% CI: 1.822, 6.130) compared to those who had not. Mothers who had employment were at higher odds of measles (AOR = 4.069; 95% CI: 1.335, 8.401) compared to those who had not (Table 4).

**Table 4:** Multivariate regression analysis of factors associated with immunization coverage among children under five years in Hargeisa, Somaliland, 2022 (n = 174)

Variables	BCG	OPV	Pentavalent	Measles
	AOR	AOR	AOR	AOR
Receiving awareness	3.887 (1.275–6.844) ^*^		11.38 (5.424–14.464)^*^	3.074 (1.822, 6.130)^*^
Employment		^−−^		4.069 (1.335–8.401) ^*^

*Significant at P-value of < 0.05

## Discussion

Vaccination is one of the most successful and cost-effective public health interventions of the well-being of the child and vaccination coverage remains an important indicator of child health outcomes in all countries. However, until recently, a number of factors have been hindering the success of providing complete vaccination in different nations for all individuals who are in need. In spite of observable progress in addressing immunization service globally, still now immunization coverage is not sufficient enough in some parts of developing countries including Somaliland. So this study tried to assess factors associated with immunization coverage among children under five years at Mohamed Mooge Health Centre in Hargeisa, Somaliland.

The rate of oral polio and measles vaccine coverage of the findings of this study was higher, compared to Somaliland health and Demographic survey 2020 [12]. This is probable due to the level of vaccine awareness of the participants of this study which is high.

The study also revealed that the probability of full childhood immunization increases as the age of the mother increases. This finding is consistent with studies in Bangladesh [14] and Kenya [15]. This can be attributed to the fact that younger mothers do not have enough experience about the importance of childhood immunization compared to those who are older. Immunization coverage was higher for those who delivered at health facilities compared to home delivery. This finding is also in line with a study conducted in Ethiopia [10] and Kenya [15]. This may be related to mothers who delivered in a health facility are more likely to obtain training on the value of immunization from health care professionals.

This study also found that the immunization coverage was higher for those who have waited less time. This study is different from the study that was done in Ethiopia [16]. This might be related to low level of health care providers, long distance to health facilities and lack of knowledge about childhood immunization among parents. This study showed that almost all of the study participants had been aware about childhood immunization. This finding is similar to a study conducted in Ethiopia [16] and Nepal [17]. The possible justification might be due to the fact that most of the study participants have access to health care providers since most of them give birth in health care facilities.

Based on multivariate regression analysis, women who were aware of childhood immunization are usually high odds of taking BCG vaccination, pentavalent and measles. This good knowledge level is consistent with other studies in Nigeria and Somalia [13, 18]. This could be due to the fact that many of the children were delivered in health facilities and obtained these vaccines before leaving the health care facilities. The findings of this study revealed that the mothers who had employment were higher odds of measles compared to those who had not. This finding is in line with a study that was done in Ethiopia [16]. This may be as occupational status of the mother improved, health seeking behaviour may perhaps increase. This in turn may have a positive impact on children’s immunization.

### Limitations of this study

This study can be interpreted in light of these limitations. Firstly, the study used a cross-sectional study design, which made it difficult to establish causal effect relationship. The fact that immunization coverage is assessed based on the report of caregivers about the doses, types and schedule of basic vaccines may lead to bias as many of them doesn’t have immunization card, which has minimized by probing them during data collection. Secondly, the study did not address mothers’ attitude towards children’s immunization and finally, the socioeconomic and parental factors that may influence completion of immunization of children. So, we suggest to conduct specific epidemiological studies other than cross sectional study that could explore additional predictors. Despite all these limitations, we hope that our findings will provide useful information to program managers and policy makers to improve immunization coverage and thereby reduce childhood morbidity and mortality in Somaliland.

## Conclusion

The findings of this study indicated that the oral polio and measles immunization coverage was higher than the regional and national coverage, but lower than the World Health Organization target. Educational level of mothers, occupation of mothers, waiting time, motivation to come back and level of awareness were statistically significant predictors of fully immunization of children. We recommended that effort should be made to increase maternal health care utilization such as antenatal care utilization. Health care providers need to conduct health education activities on the benefits and schedule of immunization to mothers, to increase the number of children to be vaccinated. The Ministry of Health should use the greatest efforts at all levels to improve their knowledge on routine immunization. As the present study revealed very important findings, further research may be conducted to find out the unaddressed determinants as well as perspective and experiences of health care providers with provision of immunization services.

## Data Availability

All the data sets used and/or analysed during the current study are available from the corresponding author (Mohamed Mussa: mohamednsg@gmail.com) upon reasonable request.

## Abbreviations

AOR: Adjusted Odds Ratio
BCG: Bacillus Calmette Guerin
CI: Confidence Interval
DOR: Dropout Rate
EPI: Expanded Program on Immunization
